# Z-Score Neurofeedback and Heart Rate Variability Training for Adults and Children with Symptoms of Attention-Deficit/Hyperactivity Disorder: A Retrospective Study

**DOI:** 10.1007/s10484-019-09439-x

**Published:** 2019-05-22

**Authors:** Kayleah M. Groeneveld, Anna M. Mennenga, Robert C. Heidelberg, Rachel E. Martin, Rachel K. Tittle, Kyle D. Meeuwsen, Linda A. Walker, Elyse K. White

**Affiliations:** 1 Neurocore, 201 Monroe Avenue NW Suite 300, Grand Rapids, MI 49503 USA; 2 Saybrook University, Alameda, CA USA

**Keywords:** Z-score neurofeedback, Heart rate variability biofeedback, QEEG, Attention-deficit/hyperactivity disorder, ADHD

## Abstract

ADHD is a common condition that causes suffering for those affected and economic loss for society at large. The current standard treatment for ADHD includes stimulant medications, which are not effective for all patients, may include side effects, and can be non-medically misused. Z-score neurofeedback (NFB) and heart rate variability (HRV) biofeedback are alternative treatment strategies that have been associated with Attention-Deficit/Hyperactivity Disorder (ADHD) symptom improvement. We utilized a retrospective pre-post study design to quantify the change in clients’ ADHD symptoms after combined NFB + HRV treatment (which included simultaneous z-score training at four sites). We also assessed whether relevant physiological measures changed in accordance with the protocol, which would be consistent with effective NFB + HRV training. Adults (n = 39) and children (n = 100) with Borderline or Clinical ADHD classifications by the Achenbach System of Empirically Based Assessment (ASEBA) received 30 sessions of NFB + HRV training. Measures were compared before and after treatment for the ASEBA, the Integrated Visual and Auditory Continuous Performance Test (IVA), ADHD medication use, HRV and breathing parameters, and quantitative electroencephalogram (QEEG) parameters. Average ASEBA Attention-Deficit/Hyperactive Problems score improved after treatment for adults and children (*p *< 0.0001), with Cohen effect sizes (*d*_*z*_) of −1.21 and −1.17, respectively. 87.2% of adults and 80.0% of children experienced improvements of a magnitude greater than or equal to the Minimal Clinically Important Difference. After treatment, 70.8% of adults and 52.8% of children who began in the ASEBA Clinical range, and 80.0% of adults and 63.8% of children who began in the ASEBA Borderline range, were classified in the Normal range. IVA scores also improved after treatment. Changes in HRV and breathing pattern after treatment were consistent with the protocol. QEEG parameters after treatment were closer to the age-based normative mean, which is consistent with effective z-score NFB training.

**Electronic supplementary material:**

The online version of this article (10.1007/s10484-019-09439-x) contains supplementary material, which is available to authorized users.

## Objective

Attention-Deficit Hyperactivity Disorder (ADHD), characterized by functional impairment due to persistent inattention and/or hyperactivity and impulsivity (Diagnostic and Statistical Manual of Mental Disorders 5 (DSM-5), (American Psychiatric Association 2013)), affects 9.5% of children and 4.4% of adults in the United States (Bloom et al. 2013; Kessler et al. 2006). ADHD behavioral symptoms and cognitive deficits have ramifications for the affected individuals, their loved ones, and society at large. Individuals with ADHD may experience difficulties with education, personal relationships, self-esteem, and quality of life (Biederman et al. 2006; Danckaerts et al. 2010; Loe and Feldman 2007; Mrug et al. 2012). Further, individuals with ADHD are over-represented in psychiatric care, substance abuse rehabilitation facilities, and prisons (Deberdt et al. 2015; Huntley et al. 2012; Mannuzza et al. 2008; Young et al. 2015). Beyond the emotional price paid by communities in reduced safety and happiness, the economic costs of ADHD exceed $140B per year in the United States (Doshi et al. 2012).

The standard treatment for ADHD is a combination of behavior therapy and stimulant medication (e.g., methylphenidate) (AAP Subcommittee Report et al. 2011). The large, NIMH-funded Multimodal Treatment of Attention Deficit Hyperactivity Disorder (MTA) study demonstrated that this combined treatment regimen, using a carefully titrated medication dose, was superior to behavior therapy alone or active control (usual treatment available in the community) in reducing ADHD symptoms in children (MTA 1999), although this regimen was not effective for all children in the combined treatment group (Swanson et al. 2001). However, this standard treatment appears to have little lasting effect on ADHD symptoms; the advantage described above of children treated with combined behavior therapy and stimulant medication dissipated less than two years after completion of the initial study (Jensen et al. 2007); further, ADHD symptoms persisted in adulthood for the combined behavior therapy and stimulant medication group (Swanson et al. 2017). There are also possible side effects with stimulant medications, including insomnia (Catala-Lopez et al. 2017), anorexia (Cerrillo-Urbina et al. 2018), and possible long-term height restriction of up to an inch (Poulton et al. 2013; Swanson et al. 2017). Further, stimulant ADHD medications can be misused (National Institute on Drug Abuse 2018), and some individuals who take them meet the criteria for stimulant abuse and/or dependence (Rabiner 2013).

Various forms of behavioral therapy, especially behavior management treatment programs, have been shown to be effective for ADHD symptom reduction in children (Evans et al. 2018). However, these therapies often do not generalize well to other aspects of the child’s life beyond the specific behaviors trained. A recent randomized clinical trial found that, for elementary school children who attended a rigorous six-week summer behavior training program, those whose parents participated in twelve hours of behavioral training performed substantially better on their homework than children in the program who took ADHD medication (Merrill et al. 2017). Although these results are encouraging, it remains to be seen whether such a program would be feasible outside of an intensive summer school setting. Therefore, the development of additional treatment strategies for ADHD, especially for those who find stimulant medication ineffective or unacceptable, is a high priority.

### Neurofeedback Therapy for Treatment of ADHD

One strategy for development of effective ADHD therapies is to target physiological differences between individuals with ADHD and controls. Neuroimaging studies demonstrate that the ADHD brain is structurally different from controls (e.g., reduced global brain size, and reduced size of the prefrontal cortex and cerebellum (Krain and Castellanos 2006)). There are also differences in function and connectivity of the ADHD brain, including reduced activity of structures and pathways involved in attention and other tasks (Bush 2010; Dickstein et al. 2006).

Neurofeedback (NFB) is a specialized form of biofeedback in which participants learn to modulate their brain activity through conscious or nonconscious control via operant conditioning. During NFB training sessions, specific components of an individual’s brain activity are continuously presented to him/her as a feedback signal in real time, extracted from electroencephalogram (EEG) recordings from the participant’s scalp. Individuals are rewarded for modifying their brain activity in accordance with a specific NFB protocol (Niv 2013; Sitaram et al. 2017; Wood et al. 2014).

NFB has been used successfully to treat individuals with ADHD (Arns et al. 2009; Lubar et al. 1995; Pigott and Cannon 2014; Sitaram et al. 2017; Thompson and Thompson 1998) and is rated as efficacious for ADHD by the Association for Applied Psychophysiology and Biofeedback (2011). Meta-analyses of randomized controlled trials have found that NFB is more effective than control for ADHD symptoms in children, as measured by ratings from individuals who were unlikely to be blinded to condition, such as parents (Micoulaud-Franchi et al. 2014; Sonuga-Barke et al. 2013). Some meta-analyses have also found NFB more effective than control for ADHD symptoms as rated by individuals who were possibly blinded, such as the child’s teacher (Micoulaud-Franchi et al. 2014; Riesco-Matias et al. 2019), though not all studies have found this (Cortese et al. 2016). A recent meta-analysis (Van Doren et al. 2018) compiled the results from ten randomized controlled trials that included long-term follow-up for the effects of NFB on ADHD symptoms in children. They found that NFB was superior to “non-active” control for treatment of both hyperactivity/impulsivity and inattention symptoms of ADHD, and that these effects were maintained for 6–12 months after the completion of NFB treatment. They further found that NFB was equally as effective as “active treatment” (medication or psychotherapy) for hyperactivity/impulsivity symptoms of ADHD, both during treatment and at follow-up at 6–12 months after treatment. Active treatment was initially more effective than NFB only for inattention symptoms of ADHD, but this advantage was not maintained at follow-up, when NFB was equally as effective as active treatment.

A recent study demonstrated that NFB may be an effective treatment for ADHD symptoms in adults, as well as children, with the effects lasting at least 6 months after the conclusion of NFB treatment (Mayer et al. 2016). Further, NFB may lead to changes in brain structure. An NFB protocol designed to improve attention in a small group of healthy university students was associated with structural changes in brain regions involved in sustained attention, including increases in gray matter volumes and increased fractional anisotropy in white matter pathways; these changes were discerned by techniques of magnetic resonance imaging, including diffusion tensor imaging (Ghaziri et al. 2013).

Z-score NFB is a relatively new method that was first proposed by Thatcher in the late 1990s (Thatcher 1998), with the first clinical reports published in 2007 and 2008 (Collura et al. 2010). Z-score NFB uses operant conditioning to train oscillatory dynamics found in the EEG toward a state characterized as more normal, based on metrics derived from a normative reference database (Thatcher and Lubar 2009). These metrics originate from a collection of quantitative EEG (QEEG) recordings of healthy individuals that are matched in age with the trainee. The z-score for a particular QEEG metric represents how many standard deviations the individual’s observed value is from the average value for his/her age-matched reference group. The goal of z-score NFB is to simultaneously train the z-scores for multiple brain oscillation metrics toward z-score = 0, the center of the age-matched normal distribution, in real time (Collura et al. 2010; Thatcher and Lubar 2009).

Currently, there are few studies of z-score NFB available in the literature (Coben et al. 2019). It was demonstrated in a small study that participants’ QEEG metrics moved closer to average values after z-score NFB, compared to before treatment (Krigbaum and Wigton 2015). Furthermore, these changes were associated with clinical symptom improvement for several different psychological conditions in fewer sessions than would be expected for traditional NFB. Z-score NFB has also been associated with improvement of insomnia symptoms (Hammer et al. 2011) and ADHD (Wigton and Krigbaum 2015) in small studies. Therefore, z-score NFB is a promising intervention strategy to reduce ADHD symptoms.

### Heart Rate Variability Biofeedback Therapy for Treatment of ADHD

Research has shown that individuals with ADHD also differ from controls in specific measures of the variability of time between heartbeats (their heart rate variability, HRV; (Griffiths et al. 2017; Rash and Aguirre-Camacho 2012; Rukmani et al. 2016)). HRV is thought to represent a physiological index of sympathetic and parasympathetic nervous system influences on the heart, and reduced HRV is associated with many negative health effects, including increased risk of mortality (Shaffer et al. 2014). HRV biofeedback therapy trains individuals to control respiratory sinus arrhythmia (RSA) in order to increase variability in their heart rate (Lehrer and Gevirtz 2014). This therapy has been used as an adjunctive treatment for psychological problems such as stress, depression, anxiety, and post-traumatic stress disorder, with promising results (Gevirtz 2013). Lloyd and colleagues administered HRV biofeedback therapy to children with ADHD in a small randomized controlled trial (Lloyd et al. 2010); they found that HRV biofeedback reduced several ADHD problem behavior symptoms. Therefore, HRV biofeedback also represents a promising, non-pharmaceutical treatment strategy for ADHD.

### Combined Z-Score Neurofeedback + Heart Rate Variability Biofeedback for ADHD

Based on the promising NFB and HRV strategies for treatment of ADHD described above, along with the need in the field for an effective, non-pharmaceutical therapy, we have developed a combined protocol of z-score NFB and HRV biofeedback (NFB + HRV). The current study seeks to assess and quantify the changes in clients’ ADHD symptoms after treatment with NFB + HRV. Before and after treatment, clients were evaluated for ADHD symptoms and behavior, ADHD medication use, physiological parameters of HRV, breathing rate, and QEEG. These pre- and post-treatment measurements were compared to evaluate whether, and the extent to which, ADHD symptoms improved after NFB + HRV treatment. We also evaluated whether the physiological parameters were significantly changed after treatment in accordance with the protocol, which would be consistent with effective NFB + HRV training.

## Materials and Methods

The NFB + HRV therapy sessions described in this study were performed by a private center that provides EEG NFB and Biofeedback therapies at locations in Michigan and Florida, USA. The center provides NFB and HRV biofeedback therapy for clients presenting with a variety of symptoms, including ADHD, anxiety, depression, memory concerns, migraines, sleep disturbances, and stress. Permission for this study was obtained from the New England Independent Review Board (IRB). This study is purely retrospective, rather than prospective. An IRB Privacy Board Waiver of Consent for retrospective studies was obtained for data analysis of clients who began a thirty-session NFB + HRV treatment program on or after February 15, 2017 that was completed by November 15, 2017, with all personal health information identifiers removed.

Both before and after the 30 sessions of NFB + HRV, all clients included in this study took behavioral assessment tests, underwent a full-cap (19-electrode) QEEG assessment, completed an HRV and breathing rate assessment, and listed all current medications (including the frequency and dose taken for each drug). This information was used to compare client ADHD symptoms, performance on an objective attention task, physiological characteristics, and medication use before and after treatment.

### Assessments

#### Behavioral Assessments

Two behavioral assessment tools were utilized in this study: the Achenbach System of Empirically Based Assessment (ASEBA) and the Integrated Visual and Auditory Continuous Performance Test (IVA). Each assessment tool was administered to clients both before and after the thirty-session NFB + HRV treatment protocol. These tools were also used to define the sample of clients included in the present study (see Client Demographics section). ADHD medication use was also assessed.

##### ASEBA

The ASEBA symptom checklist was utilized to classify and quantify the severity of symptoms associated with ADHD and comorbid disorders. The Adult Self-Report (ASR) symptom checklist (Achenbach and Rescorla 2001) was administered to adult clients. For clients under the age of 18, the Child Behavioral Checklist (CBCL; Achenbach and Rescorla 2003) was completed by a parent/guardian. As self-report and parent/guardian-report assessments, the ASEBA tools provide a subjective measure of ADHD symptom severity.

The ASEBA DSM-5 Oriented AD/H Problems subscale was the primary outcome measure utilized in this study. The output of the ASEBA scales is a *T* score that quantifies the degree and number of symptoms for each behavioral scale. The *T* score corresponds to one of three possible conditions of increasing severity: Normal (*T* ≤ 64), Borderline (*T* = 65-69), and Clinical (*T* ≥ 70). The minimum possible *T* score as defined by ASEBA is 50 (to prevent over-interpretation of differences between scores which fall undoubtedly within the normal range, ASEBA truncates *T* scores at 50). According to the ASEBA manuals (Achenbach and Rescorla 2001;Achenbach and Rescorla 2003), individuals with a ‘Normal’ *T* score have no need of professional help, ‘Borderline’ individuals have enough problems reported to be of concern, and ‘Clinical’ individuals warrant professional help for their psychological condition. We refer to the combined group of Borderline + Clinical individuals with the term ‘Symptomatic’ in this paper.

##### IVA

The IVA (IVA + Plus, Version 2014.2, BrainTrain, Richmond, VA, USA) was conducted to quantify clients’ performance on a continuous performance task (IVA + Plus Interpretation Manual, (Sandford 2014)). The IVA is a fully objective measure of task performance. There are two primary quotient scores: The Full Scale Attention Quotient (FAQ), which provides a measure of an individual’s ability to perform under conditions of low demand, and the Full Scale Response Control Quotient (FRCQ), which provides a measure of an individual’s overall ability to regulate and provide appropriate responses. Each quotient has a normalized mean score of 100 and a standard deviation of 15. The IVA also includes two separate validity checks that are intended to determine whether individuals are responding randomly to the test stimuli. To validly interpret the FRCQ and FAQ, an individual must pass each validity check. Therefore, clients in this study with an “invalid” result for the IVA at either pre- or post-treatment were not included in the IVA score statistical analyses.

##### ADHD Medication Use

Before and after completion of the 30-session NFB + HRV treatment, clients were asked to list all current medications, along with dose and frequency. ADHD medications taken by adult and child clients in this study included the brand names: Adderall, Adderall XR, Aptensio XR, Concerta, Focalin, Intuniv, Kapvay, Metadate CD, Ritalin, Strattera, and Vyvanse.

#### Physiological Assessments

Physiological assessments taken before and after clients’ completion of 30 sessions of NFB + HRV therapy included HRV parameters, breathing rate, and QEEG/EEG.

##### Assessment of Heart Rate Variability and Breathing Rate

A blood volume pulse (BVP) sensor (Thought Technology, Montreal, Canada) reading from each client’s index finger was utilized to measure heart rate. These data were collected for a total of five minutes to provide for a minimum standard short-term recording (The Task Force Report 1996). For all initial and final assessments, a ProComp5 amplifier was used, while individual sessions used a ProComp Infiniti, both with Biograph software (Thought Technology; Biograph software version 6.0.4 was used throughout the study). A power spectrum was formed from inter-beat intervals derived using a high cutoff of 2000 ms and a low cutoff of 300 ms on the raw signal data. The density (in ms^2^/Hz) of high-frequency (HF; 0.15–0.4 Hz), low-frequency (LF; 0.04–0.15 Hz), and very-low-frequency (VLF; 0.016–0.04 Hz) domains were collected, and they were expressed as percent power of each frequency band over the whole range collected (0.016–0.500 Hz).

A strain gauge respiration belt (Resp-Flex/Pro, Thought Technology) was utilized to measure respiration properties. The belt was placed around the waist of each client at the umbilicus level. The same five-minute interval used to assess HRV measurements was utilized to calculate breaths per minute.

##### Assessment of Electroencephalographic Data

EEG activity was collected with a standard protocol using a Neuron-Spectrum-3 amplifier (Neurosoft, Ivanovo, Russia) at 19 electrode locations (FP1, FP2, F3, FZ, F4, F7, F8, C3, CZ, C4, T3, T4, T5, T6, P3, PZ, P4, O1 and O2), according to International 10–20 system standards. Electro-cap surgical style caps (Electro-Cap International, Eaton, OH) were fitted according to head circumference, and electrode reservoirs were filled with Electro-Gel (Electro-cap International). EEG data were collected using Neuroguide collection software (Applied Neuroscience, Inc., Largo, FL), using a sampling rate of 500 Hz and keeping impedance at each site under 10 kΩ.

Two five-minute EEG recordings were collected for each assessment, one with eyes closed (EC) and one with eyes open (EO), both utilizing a linked-ears reference. The Neuroguide software (Applied Neuroscience, Inc.) was used for post-processing of the raw EEG signal, including artifact removal and conversion of the signal into frequency-based measures of absolute power and relative power in 1 Hz bins from 1–30 Hz at every location. Connectivity measures of coherence and phase lag were calculated among pairwise combinations of electrodes (Thatcher 2012).

Power and connectivity measurements were compared to the Applied Neuroscience Lifespan EEG Normative Database (Applied Neuroscience, Inc.). Variation from normed database means was expressed in z-score format, or the number of standard deviations away from the mean, at each of, or between, the 19 electrode locations. The z-scores were visually represented via “brain maps” and used to guide NFB training protocols. Although brain maps were created and consulted for both EC and EO conditions, only EC data were used for statistical analysis in the present study. On average, there were 101.5 s of artifact-free data pre-treatment and 116.6 s of data post-treatment for clients in this study.

#### Client Demographics

The target population for this study was all individuals (aged 6–59) who would score in the Borderline or Clinical range for ADHD on the ASEBA AD/H Problems scale before treatment, do not have a neurodevelopmental disorder, and who would complete the full 30-session NFB + HRV program using 4-channel z-score training. The sample for this study was all clients who scored in the Borderline or Clinical range for ADHD on the ASEBA AD/H Problems scale before treatment, and who completed the full 30-session NFB + HRV program using 4-channel z-score training during a time period that was defined prior to the start of analysis. This included both adults and children. The study initially included 149 clients who started the program on or after February 15^th^, 2017, were Symptomatic at baseline based on their AD/H Problems *T* score on the ASEBA symptom checklist, completed the program on or before November 15^th^, 2017, and completed an EC QEEG assessment before and after the 30-session program. This initial group of individuals excluded employees and family members of employees, due to potential conflict of interest. Further exclusion criteria for this study were: total time to complete the program was fewer than six weeks or more than 24 weeks (our center recommends that clients complete the 30-session program in 12 weeks (White et al. 2017)), IVA results consistent with a potential neurodevelopmental or neurocognitve disorder (as identified by the IVA + Plus Interpretive Flowchart for ADHD, version 2014.2), and ASEBA scores that indicated the client was denying or exaggerating the existence of problems (as defined by the ASEBA, for children: a Total Problems raw score less than 3 or more than 133; for ages 18-35: less than 9 or more than 142; for ages 36–59: less than 7 or more than 100). After exclusion criteria were applied, 139 clients remained in the current analysis. These exclusion criteria removed three individuals who completed the program in more than 24 weeks, and seven who were identified by the IVA + Plus Interpretive Flowchart for potential neurodevelopmental disorders (a classification of ‘Unspecified Neurodevelopmental Disorder’ for children or ‘Mild Neurocognitive Disorder’ for adults).

The baseline demographics for all clients included in the study are presented in Table 1, including the number of children, adults, males, and females for each group. Children (*n* = 100) ranged in age from 6 to 17 (*M* = 10.6, *SD* = 2.9) and adults (*n* = 39) ranged in age from 18 to 51 (*M* = 32.1, *SD* = 11.6). Potential comorbidities were assessed by the ASEBA for Depressive Disorder, Anxiety, Avoidant Personality Disorder, Antisocial Personality Disorder, Oppositional Defiant Disorder, and Conduct Disorder (Table 2). Of the 39 adults in this study, 15 had AD/H Problems *T* scores classified in the Borderline range and 24 in the Clinical range at baseline. Of the 100 children in this study, 47 had ADHD *T* scores classified in the Borderline range and 53 in the Clinical range at baseline.

**Table 1:** Age and gender of included clients

	Adults		Children	
	*n*	Age *M* (*SD*)	*n*	Age *M* (*SD*)
Female	27	32.4 (12.3)	28	9.5 (2.3)
Male	12	31.4 (10.3)	72	11 (3)
Total	39	32.1 (11.6)	100	10.6 (2.9)

**Table 2:** ASEBA-identified potential comorbidities for all clients included in this study

	Adults *N* = 39		Children *N* = 100	
	*n*	*%*	*n*	*%*
Depressive disorder	36	92.3	55	55.0
Anxiety disorder	18	46.2	57	57.0
Avoidant personality disorder	20	51.3	–	–
Antisocial personality disorder	13	33.3	–	–
Oppositional defiant disorder	–	–	50	50.0
Conduct disorder	–	–	36	36.0

Some clients were potentially comorbid with more than one disorder. For this reason, the percentages do not sum to 100%

*ASEBA* Achenbach system of empirically based assessment; ‘–’ indicates that the psychological disorder was not evaluated by the ASEBA for this age group; *n* number of people who were comorbid for listed psychological disorder

Although the focus of this study was on symptoms measured by the ASEBA and IVA, clients’ diagnoses, classified by the International Classification of Diseases, 10th Revision, Clinical Modification (ICD-10-CM, FY 2017); (US National Center for Health Statistics 2016), were also recorded. The diagnoses of ADHD were made by licensed mental health professionals using industry standard best practice assessment tools. These tools included standardized symptom rating scales, continuous performance testing, and clinical interview in accordance with DSM-5 diagnosis criteria. Of adults included in this study, 38.5% had a diagnosis of an ADHD subtype. Four (10.3%) were diagnosed with Combined Type, ten (25.6%) were diagnosed with Predominantly Inattentive Type, and one (2.6%) was diagnosed with Predominantly Hyperactive Type. Of the children, a majority (83%) had a diagnosis of ADHD. Fifty-three (53%) of these children were diagnosed with Combined Type, 19 (19%) were diagnosed with Predominantly Inattentive Type, and 11 (11%) were diagnosed with Predominantly Hyperactive Type (Table 3). Additional ICD-10-CM diagnoses (other than ADHD) were also recorded. Diagnoses for Adjustment Disorder, Anxiety, and/or Major Depressive Disorder (if any) for adults and children in this study are shown in Supplemental Table 1.

**Table 3:** Client ADHD subtype diagnoses

	Adults *N *= 39		Children *N *= 100	
	*n*	*%*	*n*	*%*
*Any ADHD diagnosis*^a^	15	38.5	83	83.0
Combined type	4	10.3	53	53.0
Inattentive type	10	25.6	19	19.0
Hyperactive type	1	2.6	11	11.0

^a^ICD-10-CM, FY 2017

### Interventions

All clients in this study received 30 sessions of z-score NFB + HRV training. Trained EEG technicians conducted these NFB + HRV training sessions, and they were supervised by licensed Masters of Social Work (LMSWs). All LMSWs underwent extensive training and mentoring by study author, LAW, a BCIA-certified professional (Biofeedback Certification International Alliance, Arvada, CO, USA).

#### NFB Protocol Development

4-channel z-score NFB protocols were developed by LMSWs who were either BCIA-certified themselves, or who had been trained, mentored, and supervised by BCIA-certified instructors. Training locations for each client’s protocol were selected by taking into consideration the following: functional dynamics and dysregulation in the EEG/QEEG for both EC and EO conditions; knowledge of neuroanatomy and physiology; and the nature of clients’ symptoms. The Applied Neuroscience Symptom Checklist (Applied Neuroscience, Inc.) was used as a tool for LMSWs to compare symptoms with hypothesized areas of dysregulation, in addition to their knowledge of evidence-based criteria and clinical judgement. The six sites most often utilized for training in this study were, in descending order, F3, F4, P3, P4, FZ, and PZ.

#### HRV Biofeedback Training

At the start of each NFB + HRV session, clients spent three to five minutes on slow, diaphragmatic, paced breathing, with a goal of breathing between six and eight breaths per minute while wearing a finger BVP sensor and respiration belt. The goal of this segment was to teach the client to: 1) increase the amplitude of their RSA, and 2) create an in-phase relationship between their breathing and heart oscillations. Clients observed their breaths on a monitor, along with the fluctuations of their heart rate inter-beat interval as they interacted in real time. The technicians instructed clients to adjust their breath such that the inhalation/exhalation and the variability of the heart’s inter-beat interval began to align with each other in phase. This method of breathing is intended to increase the percentage of power in the LF band of HRV (Lehrer and Gevirtz 2014). Clients viewed a bar graph and numeric indicator of the percentage of LF power they were producing and received coaching and reminders to maintain consistent breathing.

Following the three to five minutes of HRV biofeedback alone, clients began to watch a movie, which was a source of feedback for both NFB training (as described below) and continued respiratory biofeedback throughout the remainder of the training session. The finger BVP sensor was removed, but the respiration belt remained in place, at the start of the movie. Respiratory biofeedback was presented to clients as a graph displaying their breathing at the bottom of the training screen to aid in maintaining a smooth, consistent breathing pattern. When clients exceeded 8.75 breaths per minute, or their breathing pace fluctuations exceeded 35% variation between breaths, the movie screen would shrink, using a transition time of 10 seconds. Average breaths per minute and %LF (for a surrogate measure of HRV) were collected at each NFB + HRV session.

#### NFB Training

To prepare for 4-channel z-score NFB, technicians measured the client’s scalp for site placements using the standard International 10-20 system and cleaned those areas with NuPrep gel (Weaver, Aurora, CO) to remove any oils and dead skin. They attached gold cup electrodes using Ten20 conductive paste (Weaver), placing the ground electrode along the midline, and the linked reference electrodes to the ears.

Each client received a 30- to 40-minute z-score NFB session using customized site placements based on the results of their QEEG. The ProComp Infiniti (Thought Technology) and Biograph software were used to process and feed back the HRV/respiration as well as EEG signals via Neuroguide’s Dynamic Link Library (DLL; Applied Neuroscience Inc.) using a joint time–frequency analysis (JTFA) algorithm, allowing for instantaneous feedback.

NFB training was performed simultaneously with the HRV/respiratory biofeedback mechanism described in the previous section. For NFB, the reward was presented in the form of the movie playing when a set percentage of 248 variables, being measured from the 4 channels of raw EEG, were within the targeted upper and lower z-score index thresholds, described further below. The metrics monitored at the four 10-20 EEG sites were absolute power, relative power, amplitude asymmetry, coherence, phase lag, and power ratios for the following frequency bands: delta (1–4 Hz), theta (4–8 Hz), alpha (8–12 Hz), beta (12–25), beta 1 (12–15 Hz), beta 2 (15–18 Hz), beta 3 (18–25 Hz), and high beta (25–30 Hz).

The NFB training protocol required the client to keep 95% of the above 248 measurements within an adjustable range of z-score index boundaries. An 80% (± 5%) reward rate was used as an initial starting point for setting the upper and lower z-score index thresholds. Throughout the session, the client’s reward rate was monitored in real time using Biograph software. When the protocol became too “easy” (the client maintained 95% of the measurements within the set z-score threshold more than 85% of the time) or too “difficult” (the client maintained 95% of the measurements within the set z-score threshold less than 75% of the time), the targeted z-score index threshold was adjusted by trained technicians, accordingly. This reward-level guideline was augmented by therapist-monitoring of the client during the session to ensure the client was not getting bored or frustrated. Clients’ NFB progress throughout the program was monitored within the Biograph software. Average z-score values across the four channels trained were saved after every session: z-score index mean, z-score powers index mean, z-score power ratio mean, and z-score connectivity index mean. An in-depth assessment of progress was measured after 20 sessions at the four sites trained using the Biograph software. If insufficient progress was being made, the full QEEG was repeated and the NFB protocol was modified as appropriate. The ASEBA assessment was repeated at this time for roughly half of the clients to gauge behavioral improvement.

#### Psychoeducation

In addition to NFB and HRV training, clients also underwent psychoeducation. The topics covered included exercise, diet, sleep hygiene, and deep breathing (among other coping skills) contained within the center’s curriculum book entitled the “Brain Optimization Book” (unpublished). Of the clients included in this study, 13 adults and 27 children received this psychoeducation during formal meetings with a staff social worker (before or after each session) lasting approximately 20 min each. The other clients included in the study also received psychoeducation on these topics, but not in the same format of formal meetings. The psychoeducation variable (presence or absence of formal psychoeducation) was not included in analyses in this study, because non-significant regression models (one for adults and another for children) containing this variable as a potential confounder were not useful in prediction of change in AD/H Problems *T* score, the primary outcome measure. This is described in more detail in the Statistical Analysis section below.

### Statistical Analysis

The parametric statistical analyses utilized in this study were all performed with SAS^®^ Enterprise Guide, Version 7.1. Copyright, SAS^®^ Institute Inc. The line graphs were made using the software Graphpad Prism (version 5), and the stacked bar chart was made using Microsoft Excel (version 16.13.1). The box plot in Supplemental Fig. 1 was created in SAS^®^.

Although individuals of any age can have ADHD, the disorder is currently both understudied and undertreated in adults (Das et al. 2015; Ginsberg et al. 2014). For this paper, adults (ASR) and children (CBCL) were analyzed separately to further investigate the benefit that ADHD treatment can have on adults. Multiple linear regression was utilized to investigate the potentially confounding variables: gender, use of an ADHD medication at baseline, psychoeducation, region (East Michigan, West Michigan, or Florida), and age. Two separate models were created, one for each test type group: adults (ASR) and children (CBCL). For both adults and children, the models containing the aforementioned covariates were not useful in predicting the magnitude of improvement in AD/H Problems *T* scores (*p* = 0.7045, 0.6020; respectively).

P-values in this study were assessed using an experiment-wise error rate of $\alpha$ = 0.05; Bonferroni correction was used to adjust for multiple testing and calculated separately for the three types of analyses in this study: ADHD symptom outcome measures, including AD/H Problems *T* scores and IVA FAQ and FRCQ (K = 3); HRV measures (K = 4); and QEEG measures (K = 21). The Bonferroni-corrected significance level for ADHD symptom outcome measures was $\alpha_{B}$ = 0.0167 with three comparisons for each group, adults and children. The Bonferroni-corrected significance level for HRV outcome measures was $\alpha_{B}$ = 0.0125 with four comparisons for each group, adults and children. The Bonferroni-corrected significance level for QEEG outcome measures was $\alpha_{B}$ = 0.00238 with 21 comparisons. For QEEG outcomes, adults and children were combined. This is because separating them would leave adult sample sizes too small for analysis of some QEEG parameters and because the Neuroguide database, on which the z-score NFB training is based, is already age-normed.

All t-tests performed for this analysis were two-sided. For all analyses, the assumption of independence is met, yet it should be noted that a negligible proportion of clients might have been biologically related. This is a retrospective sample of all clients meeting the inclusion/exclusion criteria within the specified time range. Thus, convenience sampling was utilized.

Paired t-tests were used to assess the mean changes in AD/H Problems *T* score from pre-treatment to post-treatment. The mean changes in Standard Quotient scores for the IVA were assessed using paired t-tests for those who passed both validity checks on the IVA. Mean changes in HRV parameters and breathing rate were evaluated with paired t-tests. Clients were separated by age group (child or adult). For all t-tests the normality assumption of the paired differences was satisfied by assessing sample sizes, box plots, and histograms.

The Minimal Clinically Important Difference (MCID) for improvement in AD/H Problems *T* score from pre-treatment to post-treatment was defined as an improvement of at least three points. This value was determined in the following manner: the MCID for ASEBA score change is defined for two age ranges within each gender (for each test type, ASR or CBCL). The MCID is the Standard Error of Measure (*SE Meas*), calculated using ASEBA’s gender- and age-normed population statistics. To arrive at this value, the standard deviation is multiplied by the square root of one minus the test re-test reliability, $SE Meas = SD\left( {\sqrt {1 - Reliability} } \right)$ (Achenbach and Rescorla 2003). Because these values for MCIDs ranged between 1.35 and 2.4 (Supplemental Table 2), we selected a change of at least three points to define a clinically meaningful change.

Twenty-one z-score measurements taken during the 19-electrode QEEG assessment were compared before treatment versus after treatment, including absolute power, relative power, coherence, and phase lag for each frequency band: delta (1–4 Hz), theta (4–8 Hz), alpha (8–12 Hz), beta (12–25 Hz), and high beta (25–30 Hz). The theta/beta ratio (power of theta (4–8 Hz)/power of beta (12–25 Hz)) was also considered as a separate measure, despite the fact that the theta and beta bands were also considered individually. The theta/beta ratio is widely considered to be important for ADHD NFB training (Van Doren et al. 2018), therefore it was included in the analysis. Our statistical method for determining whether or not QEEG measures changed after treatment, in accordance with the z-score NFB protocol, was based on the method developed by (Wigton and Krigbaum 2015). Our method deviates from their work by separately calculating average z-score values for each of the 21 measurements described above and by using an absolute value transformed z-score threshold of 1.5 to identify sites of interest (SOIs). For each individual, the electrode sites trained were determined based on the QEEG of the individual, thus if more than four sites exhibited dysregulation, a training protocol could result in multiple variants of 4-channel site placements throughout the training program. The minimum number of sites trained for an individual was four and the maximum number of sites trained was nine. For each site trained for each individual, baseline absolute values of z-scores for each of these 21 measurements were defined as SOIs if they were farther than 1.5 standard deviations from the normed mean, such that their absolute value was greater than 1.5, meaning their z-score was less than -1.5 or greater than 1.5 at the pre-treatment assessment. These SOIs were recorded, such that the absolute value of the baseline z-score was recorded. Within each of these 21 measurements, the SOIs were then averaged for each individual. Therefore, in this analysis each individual had up to 21 average |z-score| values for SOIs pre-treatment. Each individual’s post-treatment average |z-scores| were calculated by averaging post-treatment z-score absolute values for corresponding SOIs that were used in the pre-treatment calculation. Paired t-tests were used to evaluate mean changes in SOIs for each parameter, such that a negative mean change of *x* would indicate that the z-scores move closer to zero by *x* z-scores on average, indicating normalization.

## Results

### ASEBA Scores

**Fig. 1:**
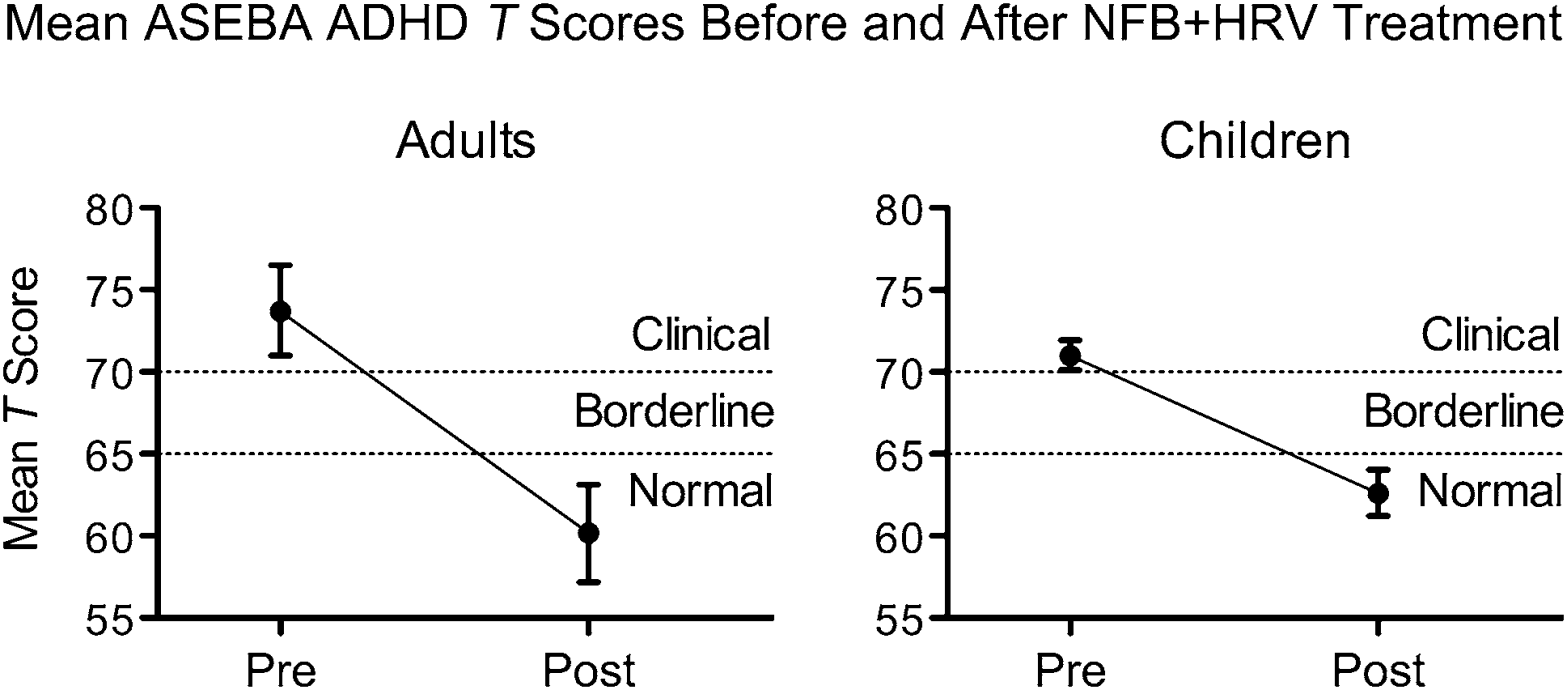
Average ASEBA AD/H Problems *T* Scores decreased after NFB + HRV treatment. For both adults (left panel) and children (right panel), average scores decreased from the Clinical range before treatment (Pre), to the Normal range after treatment (Post). These mean changes are statistically significant (Table 4). Error bars represent the 95% confidence interval. *ASEBA* Achenbach system of empirically based assessment

Before and after administration of the 30-session NFB + HRV treatment protocol, clients were evaluated for ADHD symptoms with the ASEBA DSM-oriented symptom severity checklists, ASR and CBCL. Paired t-tests were used to assess their average change in *T* score from pre-treatment to post-treatment (Table 4), and both adults and children experienced a statistically significant decrease (improvement) after treatment. Adults experienced a mean decrease of 13.6 (*SD* = 11.2, 95% CI = [−17.2, −10.0], *p* < 0.0001), and children experienced a mean decrease of 8.4 (*SD* = 7.2, 95% CI = [−9.8, −7.0], *p* < 0.0001) after NFB + HRV treatment. The effect sizes for these changes were large; *d*_*z*_ = −1.21 for adults and *d*_*z*_ = −1.17 for children. For both adults and children, the average *T* score after treatment was in the Normal range (Fig. 1).

**Table 4:** Mean changes in ASEBA AD/H problems *T* score from pre-treatment to post-treatment

	*N*	Pre	Post	Change				Change of at least the MCID *% (n)*		
		*M* (*SD*)	*M* (*SD*)	*M*_*d*_ (*SD*_*d*_) [95% CI]	*d*_z_	*t*	*p*	Improve^a^	No Δ^b^	Decline^c^
Adults	39	73.7 (8.5)	60.2 (9.2)	− 13.6 (11.2) [− 17.2, −10.0]	− 1.21	− 7.57	< .0001*	87.2 (34)	10.3 (4)	2.6 (1)
Children	100	71.0 (4.5)	62.6 (7.1)	− 8.4 (7.2) [−9.8, − 7.0]	− 1.17	− 11.74	< .0001*	80.0 (80)	15.0 (15)	5.0 (5)

*ASEBA* Achenbach system of empirically based assessment; M_d_ mean of differences; *SD*_*d*_ standard deviation of differences; *d*_*z*_ Cohen’s d for effect size of paired differences; *t* test statistic; *p p*-value for 2-sided paired t-test on differences

*Bonferroni-corrected significance level $\alpha_{B}$ = 0.0167

^a^Improve = client’s decrease in *T* score from pre to post was ≥ 3 points (the MCID)

^b^No change = client’s decrease/increase in *T* score from pre to post was < 3 points

^c^Decline = client’s increase in *T* score from pre to post was ≥ 3 points

Table 4 also includes the percentage of these adults and children who experienced a clinically meaningful improvement in ASEBA *T* score as defined by an improvement of a magnitude equal to or greater than the MCID of 3 points (see Methods). 87.2% of adults and 80.0% of children improved by at least the MCID after NFB + HRV treatment. Therefore, 12.8% of adults (5 individuals) and 20.0% of children (20 individuals) did not experience improvement in ASEBA *T* score of clinical importance after NFB + HRV treatment. Of these 5 adults, 4 (10.3% of all adults in the study) experienced no clinically important change in *T* score (the client’s decrease/increase in *T* score from pre- to post-treatment was < 3 points), and 1 (2.6% of all adults) experienced *T* score decline of clinical importance (the client’s increase in *T* score from pre- to post-treatment was ≥ 3 points). Of these 20 children, 15 (15.0% of all children) experienced no clinically important change in *T* score, and 5 (5.0% of all children) experienced *T* score decline of clinical importance.

**Fig. 2:**
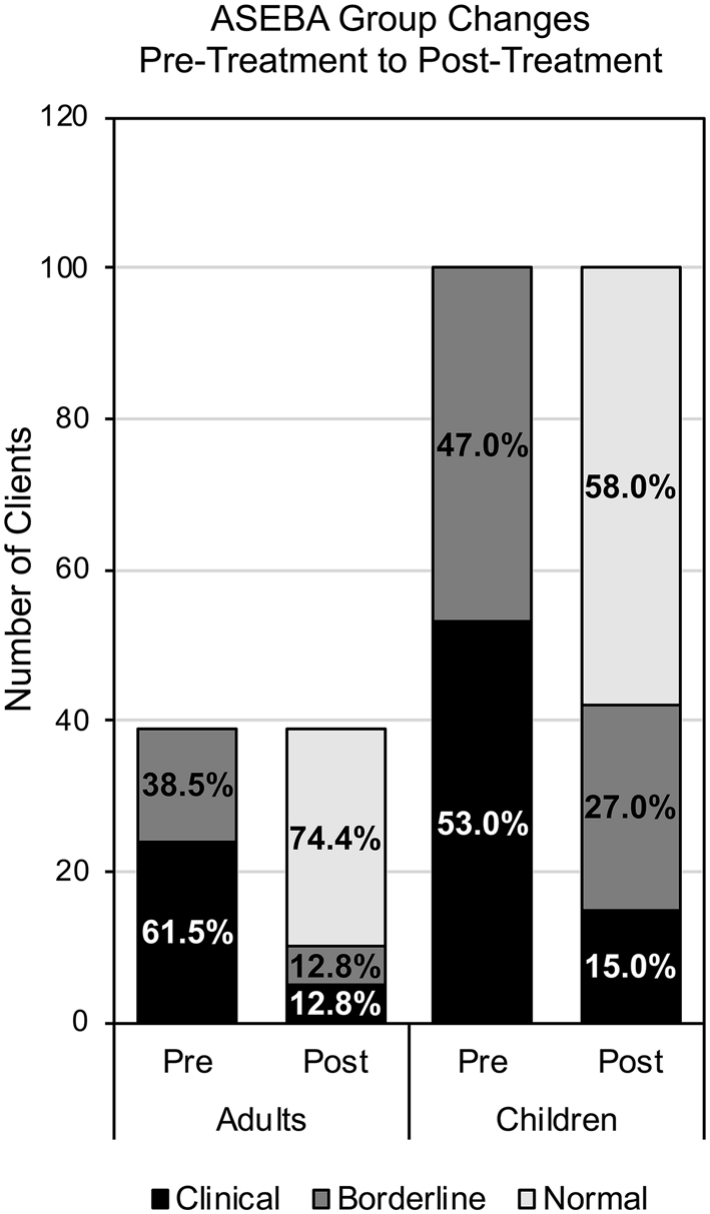
Client ASEBA ADHD classification groups before and after treatment. After NFB + HRV treatment (Post stacked bars), most adults (left) and children (right) were in the Normal ASEBA ADHD group (light gray). All clients in this study were either Borderline for ADHD (dark gray) or Clinical for ADHD (black) before treatment (Pre stacked bars). *ASEBA* Achenbach system of empirically based assessment

The changes in ASEBA ADHD classification after treatment (‘Normal’ vs ‘Symptomatic,’ which includes both ‘Clinical’ and ‘Borderline’) are listed in Table 5 and shown graphically in Fig. 2. As described, before treatment, all clients in this study were either in the Clinical or Borderline range for ADHD according to the ASEBA. After treatment, most adults (74.4%) and children (58.0%) were classified as Normal by their ADHD *T* score. More specifically, 70.8% of adults and 52.8% of children who began in the Clinical range were classified in the Normal range after treatment. 80.0% of adults and 63.8% of children who began in the Borderline range were classified in the Normal range after treatment. Not all clients experienced an improvement in ASEBA ADHD category after treatment: 16.7% of the adults and 22.6% of children who began in the Clinical range remained in the Clinical range after treatment (see Table 5 for all ASEBA ADHD classification changes).

**Table 5:** ASEBA ADHD classification group before and after NFB + HRV treatment

Group pre → Group post	Adults	Children
	*N* = 39	*N* = 100
*Clinical group at baseline*	*n = 24*	*n = 53*
Clinical → Clinical	4 (16.7%)	12 (22.6%)
Clinical → Borderline	3 (12.5%)	13 (24.5%)
Clinical → Normal	17 (70.8%)	28 (52.8%)
*Borderline group at baseline*	*n = 15*	*n = 47*
Borderline → Clinical	1 (6.7%)	3 (6.4%)
Borderline → Borderline	2 (13.3%)	14 (29.8%)
Borderline → Normal	12 (80.0%)	30 (63.8%)

*ASEBA* Achenbach system of empirically based assessment; ‘→’ indicates a change from pre- to post-treatment; *N* number of individuals in sample for age group; *n* number of individuals in either Clinical group or Borderline group at baseline for each age group; Percentages are calculated, such that the denominator is the corresponding *n*

### IVA Scores

All clients in this study also completed the IVA Continuous Performance Test both before and after 30 sessions of NFB + HRV training. Unlike the ASEBA, which relies on either self-evaluation (ASR) or parental/guardian-evaluation of behavior (CBCL), the IVA is an objective measure of task performance. The IVA was used to assess clients’ performance on two quotients relevant to attention and response control (the FAQ and FRCQ, see Methods) before and after treatment. The normative mean value for these quotients is 100, and scores between 85 and 115 are within 1.0 standard deviation of this mean. One adult and 20 children in this study failed validity checks for the IVA, suggesting that they were responding randomly to test stimuli. Therefore, these 21 clients were not included in the statistical analyses on the two IVA quotient scores presented in this section.

For adults, there was a statistically significant increase (improvement) in the mean IVA FAQ (*n *= 38, *M* = 13.2, *SD* = 26.0, $d_{z} =$ 0.51, *p* = 0.0035, 95% CI = [4.6, 21.7]). FRCQ scores were also improved after treatment, although this was not statistically significant at the $\alpha_{B}$ = 0.0167 level (*n* = 38, *M* = 7.3, *SD* = 20.1, $d_{z} = 0.36,$*p* = 0.0315, 95% CI = [0.7, 13.9]). For children, there was a statistically significant increase (improvement) in mean IVA FRCQ (*n* = 80, *M* = 6.5, *SD* = 18.9, $d_{z} = 0.34$, *p* = 0.0030, 95% CI = [2.3, 10.7]). There was also improvement in FAQ (*n* = 80, *M* = 3.4, *SD* = 18.0, $d_{z} = 0.19$, *p* = 0.0980, 95% CI = [−0.6, 7.4]), but this was not statistically significant (Table 6).

**Table 6:** Pre-treatment to post-treatment IVA quotient mean changes

	Scale	Pre	Post	Change			
		*M* (*SD*)	*M* (*SD*)	*M*_*d*_ (*SD*_*d*_) [95% CI]	*d*_z_	*t*	*p*
Adults *n* = 38	FRCQ	81.5 (27.5)	89.8 (29.1)	7.3 (20.1) [0.7, 13.9]	0.36	− 2.24	0.0315
	FAQ	78.9 (33.7)	94.2 (30.7)	13.2 (26) [4.6, 21.7]	0.51	− 3.12	0.0035*
Children *n* = 80	FRCQ	80.7 (22.3)	86.6 (21.1)	6.5 (18.9) [2.3, 10.7]	0.34	− 3.07	0.0030*
	FAQ	81.8 (24.7)	83.8 (24.6)	3.4 (18) [−0.6, 7.4]	0.19	− 1.67	0.0980

*IVA* Integrated visual and auditory continuous performance test; *n* number of individuals with valid IVA scores; *FRCQ* Full scale response control quotient; *FAQ* Full scale attention quotient; *M*_d_ mean of differences; *SD*_d_ standard deviation of differences; *d*_z_ Cohen’s d for effect size of paired differences; *t* test statistic; *p* p-value for 2-sided paired t-test on differences

*Bonferroni-corrected significance level $\alpha_{B}$ = 0.0167

**Fig. 3:**
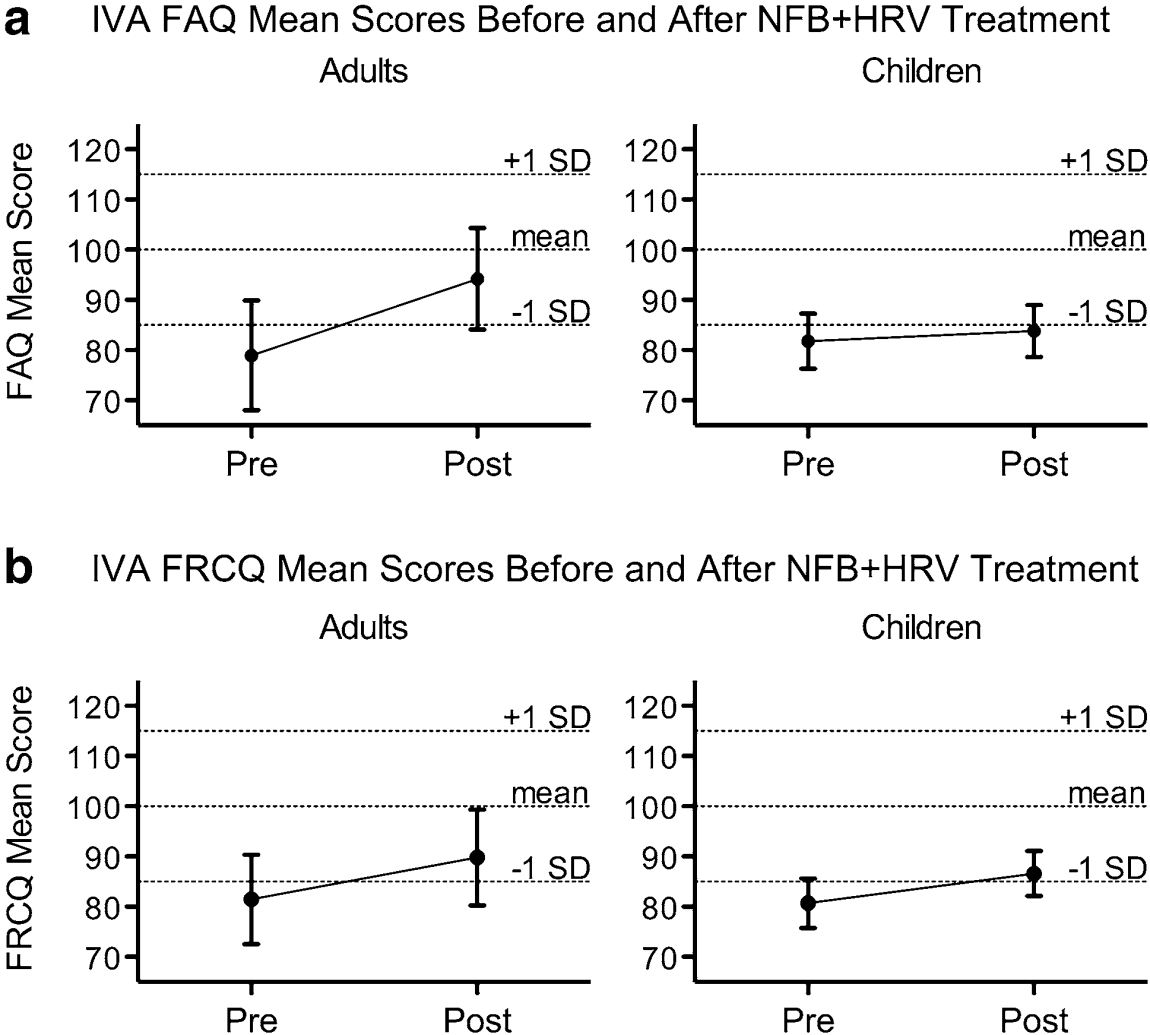
IVA Quotient Means Before and After Treatment. **a** The mean change in FAQ score after NFB + HRV treatment for adults (left panel) is statistically significant (Table 6). **b** The mean change in FRCQ score for children (right panel) is statistically significant (Table 6). Error bars represent the 95% confidence interval. Dotted horizontal lines designate the IVA normative mean for each quotient (100), as well as one standard deviation above and below this mean (85 and 115). On the IVA, a higher score indicates better performance. *IVA* Integrated visual and auditory continuous performance test; *FRCQ* Full scale response control quotient; *FAQ* Full scale attention quotient

The average quotient scores before and after treatment are displayed graphically in Fig. 3. For both adults and children, average quotient scores before treatment were greater than one standard deviation below the normative mean score (85). For adults, both mean quotient scores were within one standard deviation of the normative mean after treatment. For children, the FRCQ quotient score was within one standard deviation of the normative mean after treatment.

Supplemental descriptive statistics paralleling the 20 children who invalidated the IVA with the 80 children who did not are presented in Supplemental Table 3.

### ADHD Medication Use

Information about current ADHD medication use, dose, and frequency was collected from all clients both before and after treatment. No suggestions nor recommendations were made to clients regarding their ADHD medication regimen, and any changes made to this regimen during the program were due to external actions by the client and their prescribing physician. These medication data are displayed in Table 7 as a dichotomous variable (use of ADHD medication versus no ADHD medication used), which considers whether or not each client used ADHD medication before and after treatment.

**Table 7:** Changes in ADHD medication use from pre-treatment to post-treatment

ADHD med use Pre → ADHD med use Post	Adults	Children
	*N* = 39	*N* = 100
*No regular use of ADHD medication at baseline*	*n = 30*	*n = 56*
No Medication^a^ → Medication^a^	0% (0)	1.8% (1)
No Medication^a^ → No Medication^a^	100% (30)	98.2% (55)
*Regular use of ADHD medication at baseline*	*n = 9*	*n = 44*
Medication^a^ → Medication^a^	55.6% (5)	77.3% (34)
Medication^a^ → No Medication^a^	44.4% (4)	22.7% (10)

^a^No Medication and Medication refer strictly dichotomously to whether the client regularly used one or more ADHD medications and does not take into consideration non-ADHD medications; ‘→’ indicates a transition from pre- to post-treatment;  %’s represent the proportion of those who experienced the labeled change in that group

*N* number of individuals in sample for age group; *n* number of individuals within each age group who, at baseline, either reported regular use of ADHD medication use at baseline or did not; Percentages are calculated, such that the denominator is the corresponding *n,* and represent the proportion of those who experienced the specified pre- to post-transition within the corresponding *n*

At baseline, nine adults (23.1%) were on ADHD medication. Post-treatment, four of these individuals were no longer on ADHD medication, one reduced their daily dosage of ADHD medication, one changed their type of ADHD medication, and three had no change in their ADHD medication. No adults who were taking ADHD medication before treatment increased their daily intake after treatment. Further, no adults who did *not* take ADHD medication before treatment were taking ADHD medication after completion of treatment.

Of the children in the study, 44 (44.0%) were on ADHD medication(s) at baseline. After completing the program, ten of these individuals were no longer on ADHD medication, five reduced the daily dosage of their ADHD medication, six changed the type of their ADHD medication, 21 had no change in ADHD medication, and two increased the daily dosage of their ADHD medication. One child who was not on ADHD medication before treatment was taking ADHD medication after completion of NFB + HRV treatment.

### HRV and Breathing Rate Physiological Parameters

Physiological parameters for HRV and breathing rate were evaluated before and after treatment using four measures: %VLF band of the heart rate inter-beat interval power spectrum, %LF band, %HF band, and respiration rate (breaths per minute (BPM); Table 8). The NFB + HRV protocol in this study rewarded a breathing rate of six to eight breaths per minute. This was done to increase the proportion of the LF frequency band of HRV, and thereby decrease the percentage contribution of the other two bands.

**Table 8:** Mean changes in child and adult heart rate variability and breathing rate from pre-treatment to post-treatment

		Pre	Post	Change			
		*M* (*SD*)	*M* (*SD*)	*M*_*d*_ (*SD*_*d*_) [95% CI]	*d*_*z*_	*t*	*p*
Adults *N* = 39	%VLF	18.4 (8)	11.1 (8.3)	−7.3 (12.9) [−11.5, −3.1]	−0.57	3.55	0.0010*
	%LF	40.5 (12.2)	70.3 (19.9)	29.8 (22.6) [22.5, 37.1]	1.32	−8.23	< .0001*
	%HF	33.9 (11.2)	15.7 (12.6)	−18.2 (14.4) [−22.9, −13.6]	−1.27	7.93	< .0001*
	BPM	13.2 (2.5)	6.5 (1.3)	−6.7 (2.5) [−7.6, −5.9]	−2.67	16.65	< .0001*
Children *N* = 100	%VLF	14.3 (6.1)	9.6 (7.6)	−4.7 (10.2) [−6.8, −2.7]	−0.46	4.64	< .0001*
	%LF	34.4 (9.6)	53.2 (21.7)	18.8 (21) [14.6, 23]	0.89	−8.94	< .0001*
	%HF	43.8 (9.4)	32 (15.9)	−11.8 (16.4) [−15.1, −8.6]	−0.72	7.22	< .0001*
	BPM	13.9 (1.6)	9.3 (2.7)	−4.7 (3.2) [−5.3, −4.1]	−1.47	14.74	< .0001*

*M*_*d*_ mean of differences; *SD*_*d*_ standard deviation of differences; *d*_*z*_ Cohen’s d for effect size of paired differences; *t* test statistic; *p* p-value for 2-sided paired t-test on differences; *BPM* breaths per minute

*Bonferroni-corrected significant change ($\alpha_{B}$ = 0.0125)

Mean measures for all parameters studied were significantly changed after treatment. For adults after treatment, on average, the %VLF band decreased by 7.3 (*SD* = 12.9, $d_{z} = - 0.57,$*p* = 0.0010, 95% CI = [−11.5, −3.1]), the %LF band increased by 29.8 (*SD* = 22.6, $d_{z} = 1.32$, *p* < 0.0001, 95% CI = [22.5, 37.1]), the %HF band decreased by 18.2 (*SD* = 14.4, $d_{z} = - 1.27$, *p* < 0.0001, 95% CI = [−22.9, −13.6]), and the respiration rate decreased by 6.7 BPM to an average post-treatment value of 6.5 BPM (*SD* = 2.5, $d_{z} = - 2.67$, *p* < 0.0001, 95% CI = [-7.6, -5.9]). For children after treatment, on average, the %VLF band decreased by 4.7 (*SD* = 10.2, $d_{z} = - .46$, *p* < 0.0001, 95% CI = [−6.8, −2.7]), the %LF band increased by 18.8 (*SD* = 21.0, $d_{z} = .89$, *p* < 0.0001, 95% CI = [14.6, 23.0]), the %HF band decreased by 11.8 (*SD* = 16.4, $d_{z} = - 0.72$, *p* < 0.0001, 95% CI = [−15.1, −8.6]), and the respiration rate decreased by 4.7 BPM, to an average post-treatment value of 9.3 BPM (*SD* = 3.2, $d_{z} = - 1.47$, *p* < 0.0001, 95% CI = [−5.3, −4.1]). For both adults and children, all HRV and respiration rate physiological measures examined were significantly changed after treatment, and these changes were in the direction expected based on the specific HRV training protocol. These results are consistent with effective biofeedback training.

### QEEG Physiological Parameters

Twenty-one physiological parameters measured during the full-cap QEEG assessments before and after treatment were compared to determine whether they changed in accordance with what would be predicted from the z-score NFB protocol. Z-score NFB rewards clients when their brain oscillation parameters move closer to the age-based normative mean. As described in the Methods section, an algorithm was utilized for this study to quantify the degree to which clients’ QEEG parameters changed after z-score NFB, and, if change occurred, whether or not these parameters were closer to the normative mean after treatment (which is the goal of z-score NFB).

For all 21 QEEG parameters examined, there was a consistent trend toward the normative mean (a reduction in absolute z-score) after NFB + HRV treatment (Table 9). For 18 out of these 21 parameters, this change was statistically significant at the Bonferroni-corrected significance level for K = 21 tests of $\alpha_{B}$ = 0.00238. Coherence and phase lag were significantly closer to the normative mean after training in every frequency band examined, as was the theta/beta ratio. Effect sizes were small to medium for most parameters, with large effect sizes ranging from −1.34 to −1.66 for phase lag in all frequency bands. As is also the case for the physiological HRV and breathing rate measures described above, these results are consistent with effective training of clients’ brain oscillation parameters based on the specific z-score NFB protocol utilized.

**Table 9:** Mean changes in |z-score| QEEG parameters for averaged sites of interest (SOIs) pre-treatment to post-treatment at trained sites

Metric	Parameter	*n*	Pre	Post	Change		
			*M (SD)*	*M (SD)*	*M*_*d*_*(SD*_*d*_*)*	−*d*_*z*_	*p*
Absolute power	Delta	25	2.0 (0.4)	1.4 (0.7)	−0.612 (0.71)	0.86	0.0002*
	Theta	27	1.9 (0.3)	1.5 (0.7)	−0.419 (0.55)	0.77	0.0005*
	Alpha	33	1.8 (0.2)	1.6 (0.5)	−0.202 (0.46)	0.44	0.0157
	Beta	45	1.9 (0.4)	1.8 (0.5)	−0.143 (0.46)	0.32	0.0401
	High beta	54	2.3 (0.6)	1.8 (0.9)	−0.480 (0.81)	0.59	< .0001*
Relative power	Delta	47	2.0 (0.4)	1.7 (0.7)	−0.327 (0.63)	0.52	0.0009*
	Theta	28	1.9 (0.4)	1.6 (0.7)	−0.309 (0.53)	0.59	0.0044
	Alpha	32	1.8 (0.3)	1.5 (0.6)	−0.322 (0.54)	0.59	0.0021*
	Beta	56	1.9 (0.4)	1.6 (0.6)	−0.292 (0.53)	0.55	0.0001*
	High beta	50	2.1 (0.5)	1.6 (0.6)	−0.563 (0.57)	0.98	< .0001*
Coherence	Delta	81	2.3 (0.8)	1.4 (0.9)	−0.845 (1.09)	0.78	< .0001*
	Theta	76	2.3 (1.1)	1.4 (1.3)	−0.847 (0.99)	0.86	< .0001*
	Alpha	76	2.1 (0.7)	1.5 (1.3)	−0.606 (1.11)	0.55	< .0001*
	Beta	93	2.4 (0.8)	1.7 (1.2)	−0.701 (1.03)	0.68	< .0001*
	High beta	132	2.5 (0.7)	2.0 (1.0)	−0.518 (0.92)	0.56	< .0001*
Phase lag	Delta	94	1.9 (0.3)	0.90 (0.6)	−1.010 (0.61)	1.66	< .0001*
	Theta	83	2.0 (0.5)	1.0 (0.6)	−1.008 (0.71)	1.42	< .0001*
	Alpha	93	1.9 (0.3)	1.0 (0.6)	−0.928 (0.65)	1.43	< .0001*
	Beta	91	2.0 (0.4)	1.1 (0.7)	−0.857 (0.52)	1.66	< .0001*
	High beta	118	2.1 (0.4)	1.1 (0.7)	−0.998 (0.75)	1.34	< .0001*
Power ratio	Theta/Beta	32	1.9 (0.4)	1.5 (0.7)	−0.383 (0.54)	0.71	0.0004*

Within each parameter, for each individual, SOIs were averaged and compared pre- to post-treatment. The means of these differences are represented by M and the paired t-tests evaluate these differences

*SOI* sites of interest, z-scores of baseline values that are farther than 1.5 standard deviations from zero for the sites trained for each parameter within each metric; *Parameter* frequency band or power ratio within given metric; *n* number of people who had at least one SOI for the given parameter for the given metric; *M*_*d*_ mean of differences (change from pre to post in average distance from zero for SOIs); *SD*_*d*_ standard deviation of differences (change from pre to post in average distance from zero for SOIs); *d*_*z*_ Cohen’s d for effect size of paired differences; *p* p-value for 2-sided paired t-test on differences

*Bonferroni-corrected significant change *(*$$\alpha_{B}$$  =* 0.00238*$$)$$

## Discussion

In this study, we evaluated a novel treatment strategy combining z-score NFB with HRV biofeedback to treat clients with symptoms of ADHD. After 30 sessions of NFB + HRV, adults and children in this study experienced both statistically significant and clinically meaningful improvement in ADHD symptoms, as evaluated by the ASEBA DSM-oriented AD/H Problems scale. The magnitudes of these changes were large, with effect sizes of −1.21 for adults and −1.17 for children. 87.2% of adults experienced clinically meaningful improvements, as did 80.0% of children. Adults and children also experienced statistically significant improvements on the IVA continuous performance test, which represents an objective indicator of a client’s ability to pay attention and inhibit unwanted responses. Adults experienced statistically significant improvements in the IVA FAQ, a broad composite scale that includes measures of speed, focus, and vigilance of auditory as well as visual input (Sandford 2014). Children in this study experienced significant improvements on the IVA FRCQ, which provides a measure of an individual’s overall ability to regulate and provide appropriate responses. Before treatment, average IVA quotient scores for adults and children were all more than one standard deviation below the normative mean. After treatment, average quotient scores were within one standard deviation for adults (for both quotient scores) and for children, for the FRCQ but not the FAQ. Further, 23.1% of adults were taking ADHD medication before treatment, and 12.8% were taking ADHD medication after treatment. For children, 44% were taking ADHD medication before treatment, and 35% were taking ADHD medication after treatment. Results are in line with a growing body of literature that demonstrates that various forms of NFB constitute an effective treatment strategy for ADHD symptoms (Van Doren et al. 2018).

We have shown that clients in this study experienced physiological changes in HRV parameters, breathing rate, and QEEG parameters after treatment that are consistent with the NFB + HRV protocol utilized. Specifically, the protocol rewarded a respiration rate of six to eight breaths per minute, thus increasing the %LF component of HRV, and used 4-channel z-score NFB to train brain oscillation parameters toward the age-based normative mean from an EEG normative database (Thatcher and Lubar 2009). Z-score NFB is a promising new form of NFB to treat a number of different psychological conditions (Collura 2017), and a notable aspect of the current study is our extension, in separately examining each frequency band of each metric and the theta/beta ratio, of the algorithm developed by Wigton and Krigbaum to quantify the degree to which clients’ brain oscillation physiology changed after z-score NFB (Wigton and Krigbaum 2015). The vast majority of QEEG parameters examined at sites trained (18 out of 21) significantly changed (at the Bonferroni-corrected significance level of 0.00238) after z-score NFB treatment in the direction of the Neuroguide database normative mean, which is the goal of z-score NFB. Disregarding statistical significance, all mean changes were in the direction of the database normative mean. These data indicate normalization of the parameters, consistent with and perhaps underlying changes in reported symptom severity as well as objective improvement in continuous performance tests. A supplementary analysis of QEEG parameter change from pre- to post-treatment at sites *not* trained during NFB + HRV training is presented in Supplemental Table 4. As with the ‘sites trained’ QEEG results, the mean of differences was a negative value for every parameter examined, indicating a trend toward the normative mean. What is more, effect sizes (Cohen’s *d*_*z*_) were medium or large for all parameters and ranged from *d*_*z*_ = −0.48 for theta/beta ratio, to *d*_*z*_ = −2.20 for Phase Lag: Delta frequency band.

An elevated EEG theta/beta ratio is thought to be associated with ADHD (Lubar 1991; Monastra et al. 1999), and a device that utilizes this ratio as a biomarker has been registered by the Food and Drug Administration to aid in the diagnosis of ADHD (Food and Drug Administration 2013). Standard NFB approaches for individuals with ADHD are often focused on modifying the theta/beta ratio (Niv 2013). A recent meta-analysis demonstrated that the relationship between theta/beta ratio and ADHD is complex; elevated theta/beta ratio was not diagnostic of ADHD on the whole, but rather appears to define one ADHD subgroup (Arns et al. 2013). Our results are consistent with this; 32 out of 139 total clients (23%) began the study with theta/beta ratios at sites trained that were more than 1.5 standard deviations from the age-based normative mean. This included 22.1% of clients in this study with a Clinical ASEBA ADHD classification at baseline, and 24.5% of those with a Borderline classification at baseline. As with most of the sites-trained QEEG parameters examined in this study, the theta/beta ratio for this group of individuals was significantly closer to the normative mean after treatment (*p* = 0.0004, Table 9).

Although the vast majority of research on NFB and ADHD has examined protocols other than z-score (Coben et al. 2019), the z-score NFB protocol is a theoretically attractive choice for a heterogeneous disorder such as ADHD, because clients are trained on multiple QEEG parameters at the same time and only those for which they deviate from the mean. Further, these potential benefits for treatment of a heterogeneous disorder by z-score NFB might also extend to comorbid disorders. Clients in this study who were also in the Symptomatic range for comorbid disorders at baseline (as assessed by the ASEBA) are shown in Table 2. Supplemental Table 5 displays the proportion of adults and children for each comorbidity who were categorized by the ASEBA as Symptomatic before NFB + HRV treatment and Normal after treatment. For each comorbid disorder examined, at least 55% of those who were in the Symptomatic range before treatment were in the Normal range after NFB + HRV treatment.

After NFB + HRV treatment, clients’ HRV and breathing rate physiology changed in accordance with our biofeedback protocol. Although specific protocols differ, many studies now support the benefits of an HRV biofeedback protocol that ultimately seeks to maximize power within the LF band (Shaffer et al. 2014). These protocols have been used to treat multiple conditions, including cardiovascular and psychological disorders, with preliminary but promising results (Gevirtz 2013). The VLF component of HRV may be an intrinsic rhythm driven by the heart itself, and it also represents sympathetic nervous system activity (Shaffer et al. 2014). The HF component of HRV is thought to primarily reflect parasympathetic nervous system activity (The Task Force Report 1996). The LF component of HRV is thought to represent activity of the baroreceptors that drive blood pressure and may represent activity from both parasympathetic and sympathetic nervous system input (Billman 2013). Potential mechanisms by which power increases within the LF band might lead to psychological and physiological benefits may include strengthening of the baroreflex system and stimulation of the vagal afferent pathway (Lehrer and Gevirtz 2014).

The present study is a detailed pre-post analysis of past client outcomes, and it therefore has the limitations associated with this type of study. The experimental design was retrospective, the sample was not random, and there was no sham-control placebo group. Because there was only one treatment condition, we also could not compare the relative importance of NFB versus HRV biofeedback. Further, 20 children were excluded from the IVA pre-post analysis due to invalid test results. Supplemental descriptive statistics including baseline similarities and differences between the 20 children who invalidated the IVA (at either timepoint) versus those who did not invalidate are shown in Supplemental Table 3. Many baseline measures were similar between these two groups of children, including comorbidities, symptom severity, and ADHD medication use. For the children in our sample, the age of those who invalidated the IVA was notably younger than those who did not invalidate. This was examined by comparing the three descriptive measures of center (mean, median, and mode), Q1 and Q2 (the values deriving the interquartile range), and boxplots of the distributions of age for the groups. For all three measures of center, and both quartiles, age was lower for the group of children that invalidated the IVA. Side-by-side boxplots of age distribution can be viewed in Supplemental Fig. 1. We also observed (descriptively only, statistical significance not assessed) that mean ASEBA AD/H Problems *T* score improvement for the group that invalidated was lower (*M*_*d*_ = −5.8; *SD*_*d*_ = 7.6) than the overall mean reported in our analysis, while the mean improvement for those who did not invalidate was higher (*M*_*d*_ = −9.1; *SD*_*d*_ = 6.9) than that reported in our analysis. Note, however, that mean improvement for both of these groups was well above the MCID of three points. As our target population included all individuals aged 6-59 who would be Symptomatic for ADHD (based on the ASEBA AD/H Problems DSM-oriented scale), and children who may invalidate the IVA are included in this population, the authors felt it appropriate to analyze the total group of 100 children as a whole. Although these children were similar to the “included” children on most baseline measures, due to the age distribution of these two groups in our sample, caution should be used when generalizing IVA findings from this study to younger children.

Despite these limitations, this study utilized both ASEBA (a validated symptom severity questionnaire) and IVA (an objective performance test) neuropsychological assessment tools, considered changes in medication use, assessed physiological changes in HRV and QEEG parameters, and included a very large sample size for studies of this kind (Coben et al. 2019). This is one of the largest studies to date to examine the effects of z-score NFB on individuals with symptoms of ADHD, and, to our knowledge, it is the first of its kind to combine z-score NFB with HRV biofeedback for ADHD symptoms. The robust effect sizes and the confirmation of all expected physiological changes (including heart rate and brain oscillation dynamics) are encouraging, and increase the likelihood that the significant improvements in ADHD symptoms are due to specific effects of the NFB + HRV treatment.

## Conclusions

ADHD is a common condition in the United States (Bloom et al. 2013; Kessler et al. 2006) that causes suffering (Biederman et al. 2006; Danckaerts et al. 2010; Loe and Feldman 2007; Mrug et al. 2012) and economic loss (Doshi et al. 2012). Standard treatment for ADHD includes stimulant medications (AAP Subcommittee Report et al. 2011) that are not effective for all patients (Shim et al. 2016; Swanson et al. 2001), have side effects (Swanson et al. 2017), and carry a risk of non-medical misuse (National Institute on Drug Abuse 2018; Rabiner 2013). Alternative treatment strategies are needed for individuals with ADHD for whom stimulant medications are unacceptable or ineffective. In this retrospective pre-post design study, we demonstrated that, on average, adults and children with symptoms of ADHD improve on the ASEBA AD/H Problems DSM-oriented scale and IVA after NFB + HRV treatment. We have also shown that clients’ physiological heart rate, breathing rate, and brain oscillation parameters are changed, on average, after treatment in accordance with the training protocol, as would be expected from effective NFB + HRV training. Therefore, NFB + HRV therapy represents a promising treatment strategy for symptoms of ADHD.

## Electronic supplementary material

Below is the link to the electronic supplementary material.

Supplementary material 1 (DOCX 54 kb)

Supplementary material 2 (TIFF 56 kb)
