# Supplementary material for: Z-Score Neurofeedback and Heart Rate Variability Training for Adults and Children with Symptoms of Attention-Deficit/Hyperactivity Disorder: A Retrospective Study
Source: Appl Psychophysiol Biofeedback. 2019 May 22;44(4):291–308. doi: 10.1007/s10484-019-09439-x (PMC6834758; doi:10.1007/s10484-019-09439-x)
Supplement: Supplementary file 1 — Supplementary material 1 (DOCX 54 kb) [file 10484_2019_9439_MOESM1_ESM.docx]

**Supplemental Table 1.** Additional client ICD-10-CM diagnoses

|  | **Adults**  ***N* = 39** | | **Children**  ***N* = 100** | |
| --- | --- | --- | --- | --- |
|  | ***n*** | ***%*** | ***n*** | ***%*** |
| Adjustment Disorder | 1 | 2.6% | 4 | 4.0% |
| Anxiety | 14 | 35.9% | 28 | 28.0% |
| Major Depressive Disorder | 23 | 59.0% | 4 | 4.0% |

*Note:* Some clients were diagnosed with more than one disorder. For this reason, the percentages do not sum to 100%. Diagnoses via ICD-10-CM, FY 2017.

**Supplemental Table 2.** Calculations Used to Define the MCID for this Study.

| **Assessment** | **Gender** | **Age group** | **SD** | **reliability** | **SD*(sqrt(1-rel))** | **Max for Test Type** |
| --- | --- | --- | --- | --- | --- | --- |
| CBCL | boys | 6--11 | 5.5 | 0.93 | 1.46 | 2 |
|  |  | 12--18 | 5.2 | 0.93 | 1.38 |  |
|  | girls | 6--11 | 5.3 | 0.93 | 1.4 |  |
|  |  | 12--18 | 5.1 | 0.93 | 1.35 |  |
| ASR | men | 18--35 | 6 | 0.84 | 2.4 | 3 |
|  |  | 36--59 | 5.1 | 0.84 | 2.04 |  |
|  | women | 18--35 | 5.9 | 0.84 | 2.36 |  |
|  |  | 36--59 | 5.2 | 0.84 | 2.08 |  |
| Total Max: |  |  |  |  |  | **3** |

**Supplemental Table 3.** Comparison of children with “Valid” IVA validity checks vs children with “Invalid” IVA validity checks

|  | **“Valid” IVA Validity Checks**  **(*N* = 80)** | | **“Invalid” IVA Validity Checks**  **(*N*= 20)** | |
| --- | --- | --- | --- | --- |
| **IVA Invalidation** | ***n* (% of 80)** | | ***n* (% of 20)** | |
| IVA Valid Pre → IVA Valid Post | 80 (100%) | | 0 (0%) | |
| IVA Valid Pre → IVA Invalid Post | 0 (0%) | | 1 (5%) | |
| IVA Invalid Pre → IVA Valid Post | 0 (0%) | | 9 (45%) | |
| IVA Invalid Pre → IVA Invalid Post | 0 (0%) | | 10 (50%) | |
|  |  | |  | |
| **Age** **Descriptive Statistics** | *M* = 11.1  *SD* = 2.9  *Median* = 11  *Mode* = 9 | *Range* = [6, 17]  *IQR* = 4  *Q1* = 9  *Q3* = 13 | *M* = 8.7  *SD* = 2.4  *Median* = 8  *Mode* = 7 | *Range* = [6, 16]  *IQR* = 3  *Q1* = 7  *Q3* = 10 |
|  |  | |  | |
| **Baseline ASEBA Symptomatic Comorbidities** | ***n* (% of 80)** | | ***n* (% of 20)** | |
| Oppositional Defiant Disorder | 42 (52.5%) | | 8 (40%) | |
| Conduct Disorder | 29 (36.25%) | | 9 (35%) | |
| Anxiety | 44 (55%) | | 13 (65%) | |
| Depressive Disorder | 46 (57.5%) | | 9 (45%) | |
|  |  | |  | |
| **ASEBA AD/H Problems *T* Scores** | ***n* (% of 80)** | | ***n* (% of 20)** | |
| Baseline Clinical Group | 42 (52.5%) | | 11 (55%) | |
| Baseline Borderline Group | 38 (47.5% ) | | 9 (45%) | |
| Symptomatic Group → Normal Group | 51 (63.75%) | | 7 (35%) | |
| % that improved by the MCID | 67 (83.75%) | | 13 (65%) | |
| Change in *T* score from pre to post | *M_d_* = -9.1*, SD_d_* = 6.9 | | *M_d_* = -5.8, *SD_d_*= 7.6 | |
|  |  | |  | |
| **ADHD Medication Status** |  | |  | |
| *On ADHD Medications at Baseline* | 45% of the 80 (36) | | 40% of the 20 (8) | |
| Medication^a^ → No Medication^a^ | 22% of the 36 (8) | | 25% of the 8 (2) | |
| Medication^a^ → Medication^a^ | 77% of the 36 (28) | | 75% of the 8 (6) | |
| *Not on ADHD Medication at Baseline* | 55% of the 80 (44) | | 60% of 20 (12) | |
| No Medication^a^ → No Medication^a^ | 100% of the 44 (44) | | 91.6% of the 12 (11) | |
| No Medication^a^ → Medication^a^ | 0% of the 44 (0) | | 8.3% of the 12 (1) | |

*ASEBA* Achenbach system of empirically based assessment; *IVA* Integrated visual and auditory continuous performance test; *IQR* Interquartile range; *Q1* Quartile 1; *Q3* Quartile 3; *M_d_* Mean difference; *SD_d_* Standard deviation of differences; ^a^No Medication and Medication refer strictly dichotomously to whether the client regularly used one or more ADHD medications and does not take into consideration non-ADHD medications; ‘→’ indicates a transition from pre- to post-treatment

**Supplemental Table 4.** Mean changes in |z-score| QEEG parameters for averaged sites of interest (SOIs) pre-treatment to post-treatment at sites not trained

|  |  |  | **Change** | |
| --- | --- | --- | --- | --- |
| **Metric** | **Parameter** | ***n*** | ***M_d_ (SD_d_)*** | ***-d_z_*** |
| **Absolute Power** | Delta | 46 | -0.5607 (0.702) | 0.80 |
|  | Theta | 40 | -0.4097 (0.523) | 0.78 |
|  | Alpha | 52 | -0.2568 (0.443) | 0.58 |
|  | Beta | 63 | -0.3088 (0.498) | 0.62 |
|  | High Beta | 67 | -0.6416 (0.753) | 0.85 |
| **Relative Power** | Delta | 72 | -0.5091 (0.612) | 0.83 |
|  | Theta | 50 | -0.3863 (0.449) | 0.86 |
|  | Alpha | 49 | -0.3945 (0.525) | 0.75 |
|  | Beta | 66 | -0.4205 (0.593) | 0.71 |
|  | High Beta | 67 | -0.6563 (0.543) | 1.21 |
| **Coherence** | Delta | 139 | -0.7105 (0.605) | 1.17 |
|  | Theta | 139 | -0.6526 (0.563) | 1.16 |
|  | Alpha | 139 | -0.628 (0.536) | 1.17 |
|  | Beta | 138 | -0.6692 (0.646) | 1.04 |
|  | High Beta | 139 | -0.5234 (0.669) | 0.78 |
| **Phase Lag** | Delta | 126 | -1.0889 (0.496) | 2.20 |
|  | Theta | 123 | -1.0303 (0.494) | 2.09 |
|  | Alpha | 120 | -1.0255 (0.598) | 1.71 |
|  | Beta | 120 | -0.8152 (0.513) | 1.59 |
|  | High Beta | 134 | -0.7356 (0.413) | 1.78 |
| **Power Ratio** | Theta/Beta | 52 | -0.3303 (0.694) | 0.48 |

Within each parameter, for each individual, SOIs were averaged and compared pre- to post-treatment. The means of these differences are represented by *M_d_* and the paired t-tests evaluate these differences.

*SOI* Sites of interest, z-score of baseline values that are farther than 1.5 standard deviations from zero for the sites trained for each parameter within each metric; *Parameter* frequency band or power ratio within given metric; *n* number of people who had at least one SOI for the given parameter for the given metric; *M_d_* mean of differences (change from pre to post in average distance from zero for SOIs); *SD_d_* standard deviation of differences (change from pre to post in average distance from zero for SOIs); *d_z_* Cohen’s d for effect size of paired differences

**Supplemental Table 5.** Clients categorized by the ASEBA as symptomatic before NFB+HRV treatment and normal after treatment for potentially comorbid disorders

|  | **Adults** | | **Children** | |
| --- | --- | --- | --- | --- |
|  | ***N*** | ***n (%)*** | ***N*** | ***n (%)*** |
| Depressive disorder | 36 | 25 (69.4%) | 55 | 36 (65.5%) |
| Antisocial personality disorder | 12 | 10 (83.3%) | -- | -- |
| Anxiety disorder | 18 | 15 (83.3%) | 57 | 39 (68.4%) |
| Avoidant personality disorder | 20 | 11 (55%) | -- | -- |
| Oppositional defiant disorder | -- | -- | 50 | 34 (68.0%) |
| Conduct disorder | -- | -- | 36 | 22 (61.1%) |

*Note:* some clients were comorbid for more than one condition, thus the *N’s* and *n’s* for each condition are not mutually exclusive.

ASEBA Achenbach system of empirically based assessment; -- indicates that the psychological disorder was not evaluated by the ASEBA for this age group; *N* number who were Symptomatic for the psychological disorder at baseline; *n* number who were classified as Normal by the ASEBA for the psychological disorder after treatment; *n (%)* % relative to the row *N* **Supplemental Figure 1. Comparison of Age Distribution of Children with “Valid” IVA Validity Checks and Children with “Invalid” IVA Validity Checks**Age = Age of client at time of baseline IVA assessment ‘Invalidated IVA’ group represents 20 children, 19 of whom invalidated the IVA at baseline and 1 (age 9) who invalidated after program completion.

‘Did not invalidate IVA’ group represents 80 children who did not invalidate the IVA at either time point.

The º symbol located at x = 16 for the ‘Invalidated IVA’ group denotes an outlier, defined as a point which falls more than 1.5 times the interquartile range (IQRinvalid = 3) above the third quartile (Q3invalid = 10).
