# Supplementary figures and images for: Z-Score Neurofeedback and Heart Rate Variability Training for Adults and Children with Symptoms of Attention-Deficit/Hyperactivity Disorder: A Retrospective Study
Source: Appl Psychophysiol Biofeedback. 2019 May 22;44(4):291–308. doi: 10.1007/s10484-019-09439-x (PMC6834758; doi:10.1007/s10484-019-09439-x)

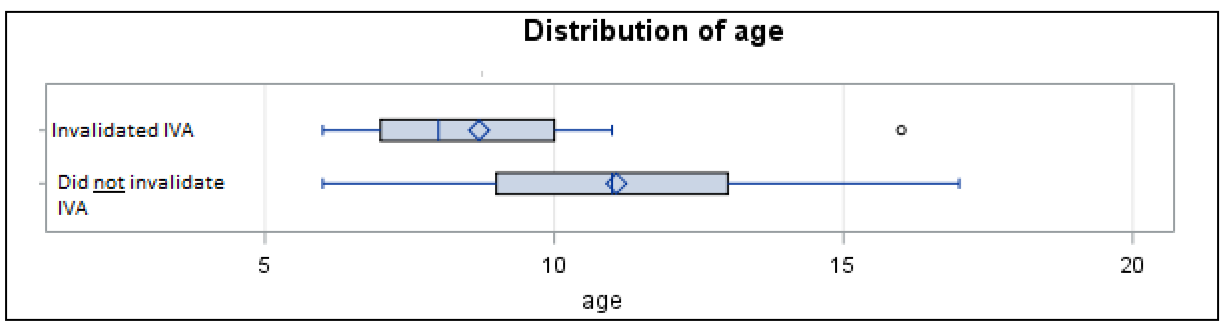

Supplement: Supplementary file 2 — Supplementary material 2 (TIFF 56 kb) [file 10484_2019_9439_MOESM2_ESM.tif]
